# Pitfalls in efficacy testing – how important is the validation of neutralization of chlorhexidine digluconate?

**DOI:** 10.1186/1476-0711-7-20

**Published:** 2008-12-02

**Authors:** Mirja Reichel, Peter Heisig, Günter Kampf

**Affiliations:** 1 Bode Chemie GmbH & Co, KG, Scientific Affairs, Melanchthonstr. 27, 22525, Hamburg, Germany; 2 Department of Pharmaceutical Biology and Microbiology, Institute of Pharmacy, University of Hamburg, Hamburg, Germany; 3 Institute for Hygiene and Environmental Medicine, Ernst Moritz Arndt University, Walther-Rathenau-Str. 49a, 17475, Greifswald, Germany

## Abstract

**Background:**

Effective neutralization of active agents is essential to obtain valid efficacy results, especially when non-volatile active agents like chlorhexidine digluconate (CHG) are tested. The aim of this study was to determine an effective and non-toxic neutralizing mixture for a propan-1-ol solution containing 2% CHG.

**Methods:**

Experiments were carried out according to ASTM E 1054-02. The neutralization capacity was tested separately with five challenge microorganisms in suspension, and with a rayon swab carrier. Either 0.5 mL of the antiseptic solution (suspension test) or a saturated swab with the antiseptic solution (carrier test) was added to tryptic soy broth containing neutralizing agents. After the samples were mixed, aliquots were spread immediately and after 3 h of storage at 2 – 8°C onto tryptic soy agar containing a neutralizing mixture.

**Results:**

The neutralizer was, however, not consistently effective in the suspension test. Immediate spread yielded a valid neutralization with *Staphylococcus aureus, Staphylococcus epidermidis *and *Corynebacterium jeikeium *but not with *Micrococcus luteus *(p < 0.001) and *Candida albicans *(p < 0.001). A 3-h storage period of the neutralized active agents in suspension resulted in significant carry-over activity of CHG in addition against *Staphylococcus epidermidis *(p < 0.001) and *Corynebacterium jeikeium *(p = 0.044). In the carrier test, the neutralizing mixture was found to be effective and non toxic to all challenge microorganisms when spread immediately. However, after 3 h storage of the neutralized active agents significant carry-over activity of CHG against *Micrococcus luteus *(p = 0.004; Tukey HSD) was observed.

**Conclusion:**

Without effective neutralization in the sampling fluid, non-volatile active ingredients will continue to reduce the number of surviving microorganisms after antiseptic treatment even if the sampling fluid is kept cold straight after testing. This can result in false-positive antiseptic efficacy data. Attention should be paid during the neutralization validation process to the amount of antiseptic solution, the storage time and to the choice of appropriate and sensitive microorganisms.

## Background

Different alcohols such as propan-1-ol, propan-2-ol and ethanol as well as chlorhexidine digluconate (CHG) have been used as effective antiseptic agents for many years.

One of the main properties of CHG is its residual antimicrobial activity which is beneficial in skin antiseptics used for catheter care [1] but not in hand hygiene [2]. Combinations of CHG and an alcohol have an advantage over single compounds. For example, the benefits of combining these substances include the immediate reduction of bacterial density by alcohols and the prolonged antibacterial effect of CHG.

The use of CHG, which has been shown to be superior to other skin antiseptics in preventing catheter-related bloodstream infections [1], is recommended by the Centers for Disease Control and Prevention (CDC) for skin antisepsis of catheter insertion sites [3]. The use of 2% CHG-based preparations before catheter insertion and during dressing changes is "strongly recommended for implementation and strongly supported by well-designed experimental, clinical, or epidemiologic studies" [3]. This recommendation is based on various studies which show that the incidence of catheter-associated primary bloodstream infection can be significantly reduced when CHG is used for treatment of the catheter insertion site [1].

The "residual effect" of CHG in hand hygiene preparations, however, is not measured in the same way. This is commonly determined by measuring the reduction of bacterial density, e.g. on fingertips [4,5] or hands [6].

In order to achieve valid results of practical relevance on the efficacy of skin antiseptics and hand disinfectants, they are usually tested *in vitro *and under practical conditions in healthy volunteers.

The main objective of efficacy tests is to determine the number of surviving microorganisms after a specific contact time measured at a defined time point. Therefore, the continuation of the antimicrobial effects of an antiseptic after the chosen contact time must be excluded by complete inactivation of the antimicrobial substances at this specific time. This process results in quenching the antimicrobial activity of a formulation and is defined as neutralization by the American Society for Testing and Materials (ASTM) [7].

In efficacy studies without neutralization in the sampling fluid, the number of surviving microorganisms is often remarkably low suggesting a high efficacy of CHG [8-10]. However, this effect can not be explained by efficacy during the exposure time but is achieved by continuous antimicrobial activity after the exposure time [9]. That is why the credibility of antimicrobial efficacy results depend largely on the performance of validated neutralization [10], especially when non-volatile active agents like CHG are tested [9,11].

One crucial point in neutralization evaluation and validation is the comparability of the *in vitro *neutralizer test and the effectiveness evaluation under practical conditions [12]. In this process all relevant critical parameters of the antiseptic test should be taken into the neutralizer validation process such as different and relevant types of microorganisms and carriers as well as storage conditions used in the efficacy test.

Even though the recently published guidelines for antiseptic efficacy tests require different forms of neutralization [13,14] and neutralization validation [15], many of the efficacy data from published studies were obtained either without adequate neutralization, without validated neutralization or without specification of the neutralizing process (e.g. storage time and temperature) and the validation status [2,16,17].

For the validation of neutralization, a European norm [18] as well as an ATSM standard [7] are available. Both of these methods focus on the effectiveness of the neutralizer on the one hand and the toxicity to test organisms on the other. Both methods describe different forms of neutralization.

The ASTM E 1054-02 method encourages the use of all microorganisms of the efficacy test also in the neutralization assay. The investigator is allowed to select the appropriate, representative microorganisms for the efficacy test [7].

In contrast, the European norm (EN) 13727 requires the use of four different test organisms (*Escherichia coli *(k12) NCTC 10538, *Pseudomonas aeruginosa *ATCC 15442, *Staphylococcus aureus *ATCC 6538 and *Enterococcus hirae *ATCC 10541) which may not always be appropriate for the efficacy evaluation [18]. Due to the fact that this norm is designed for *in vitro *suspension tests and not for *in vivo *tests, and that different microorganisms show different sensitivity to the carry-over effect of antiseptics, this choice of species may not be suitable for the validation of neutralization for specific tests such as skin antiseptics. However, there is no other comparable standard available besides the above mentioned ASTM method, which is obligatory for the ASTM test of skin antiseptics [19] and which includes the validation of neutralization.

In the present study, neutralization validation by chemical inhibition using an alcoholic solution containing 2% CHG was carried out using 5 bacterial species relevant for skin antisepsis and which were, with the exception of *Staphylococcus aureus*, not part of the EN.

The aim of this study was to determine whether the validity of neutralization was influenced by the type of test (suspension test versus carrier test), by the type of microorganism or by different storage times of the neutralized antiseptic solution containing the particular microorganism.

## Methods

### Antiseptic solution

A solution containing 89.5% (v/v) propan-1-ol and 2% (w/w) CHG, manufactured by Bode Chemie GmbH & Co. KG, Hamburg, Germany, was used in this study. The concentrations of the active agents were checked prior to validation by gas chromatography (propan-1-ol) and ultraviolet spectroscopy (CHG). The allowed tolerance was defined as 1% relative to the propan-1-ol concentration and 3% relative to the concentration of CHG.

### Test organisms

Five clinically relevant species, all found on human skin, were tested separately: *Staphylococcus aureus *ATCC 6538; *Staphylococcus epidermidis *ATCC 12228, *Micrococcus luteus *ATCC 4698, *Corynebacterium jeikeium *ATCC 43734, and *Candida albicans *ATCC 10231.

### Neutralizing agents

The following neutralizing agents were used:

• In tryptic soy broth: 3% polysorbate 80, 0.3% lecithin, 0.1% L-histidine, 0.5% sodium thiosulfate, 3% saponine and 1% ether sulfate. The suitability of these agents has been described previously [2]

• In tryptic soy agar: 0.1% L-histidine, 0.3% lecithin and 3% polysorbat 80.

### Test procedure

Experiments were carried out according to ASTM E 1054-02 "Neutralization Assay with Recovery on Solid Medium" [7].

Inocula were prepared by transferring microorganisms from stock cultures onto tryptic soy agar plates containing neutralizer which were incubated for 24 – 48 h at 37 ± 2°C. A second passage was generated under similar conditions. Microorganisms were suspended in saline peptone water, transferred into sterile flasks and stirred for about 10 minutes. Aliquots of selected dilutions were used for the test to ensure a microbial density of about 30 – 100 colony-forming units (CFU) in 1 mL of sampling fluid based on an initial volume of 5 mL.

#### Neutralizer effectiveness test

The neutralizer effectiveness tests were performed in two ways: with a rayon swab carrier and in suspension without carrier with 5 mL of tryptic soy broth containing the neutralizing agents.

• Carrier test:

The rayon swab carrier (BBL CultureSwab, Sterile, Becton Dickinson GmbH, Heidelberg, Germany) was placed in 2.5 mL of the antiseptic solution; after 20 s the swab was taken out of the antiseptic solution and the tip of the saturated swab was broken off and placed into the broth (amount of the antiseptic solution on the saturated swab: approx. 0.14 mL, measured as the difference in volume between a dry swab and a completely saturated swab)

• Suspension test:

0.5 mL of the antiseptic solution was added to 5 mL of broth.

#### Neutralizer toxicity test

The test organism was added to 5 mL of saline peptone water containing the neutralizing mixture.

#### Organism viability test

The test organism was added to 5 mL of saline peptone water without the neutralizing mixture.

#### Material control test

The test organism was added to 5 mL of the antiseptic solution.

In all tests, the sample was mixed and within 5 s an aliquot of the test organism was added.

After addition of the test organism, the samples were mixed for 30 s and:

**1**. within one minute, aliquots were plated in duplicate

**2**. after 3 h storage at 2 – 8°C, aliquots were plated in duplicate (to simulate the longest possible exposure period in the antimicrobial efficacy test).

Tryptic soy agar plates containing the neutralizing agents were used in the effectiveness tests as well as in the toxicity tests. For the detection of organism viability and material control, tryptic soy agar without neutralizer was used as a solid medium. After incubation (aerobically, 48 h at 37 ± 2°C) the number of CFU was counted, the mean number of CFU in both plates was calculated and transformed to a log_10_-value.

### Statistical analysis

Each experiment was carried out in triplicate. The results were evaluated as arithmetic means of the log_10_-values. An analysis of variance between the tests (neutralizer toxicity, material control, organism viability and neutralizer effectiveness) was performed for each microorganism. In the event of a significant difference (p < 0.05) being observed, a post-hoc test (Tukey-HSD) was carried out. Two criteria had to be met to categorize the neutralization as invalid:

The difference in the means between the neutralizer effectiveness test and the organism viability test must be

1: significant in post-hoc analysis, and

2: > 0.2 log_10_-steps (defined limit of biological relevance)

Besides the direct comparison with the organism viability test, we calculated the difference between the CFU [log_10_] after 3 h storage and that of the immediate spread samples and compared the means of these differences for each replicate and each microorganism.

## Results

The neutralizing agents showed no toxicity even after 3 h storage time. The antiseptic solution was active against all challenge microorganisms after a short time as well as after 3 h of storage (Table 1). In all cases, no microorganisms survived the material control test which resulted in significant differences in comparison to the organism viability test (p < 0.001) indicating that the antiseptic solution had strong activity against all five organisms tested.

**Table 1:** Determination of neutralizer toxicity, material antiseptic activity and organism viability according to ASTM E 1054-02.

Storage time	Test organism exposed to (name of the test)	*Staphylococcus * *aureus*	*Staphylococcus * *epidermidis*	*Micrococcus * *luteus*	*Corynebacterium * *jeikeium*	*Candida * *albicans*
< 1 min	neutralizer in saline peptone water (neutralizer toxicity test)	1.86 ± 0.05	1.81 ± 0.03	1.77 ± 0.01	1.82 ± 0.02	2.05 ± 0.01
	antiseptic solution (material control test)	0*	0*	0*	0*	0*
	saline peptone water (organism viability test)	1.90 ± 0.05	1.84 ± 0.07	1.74 ± 0.03	1.79 ± 0.02	2.02 ± 0.03

3 h	neutralizer in saline peptone water (neutralizer toxicity test)	1.83 ± 0.02	1.81 ± 0.02	1.72 ± 0.03	1.89 ± 0.08	2.05 ± 0.06
	antiseptic solution (material control test)	0*	0*	0*	0*	0*
	saline peptone water (organism viability test)	1.88 ± 0.06	1.81 ± 0.02	1.73 ± 0.05	1.77 ± 0.08	2.03 ± 0.02

Mean and SD of the number of CFU (log_10_) when exposed to the neutralizer in saline peptone water, the antiseptic solution or saline peptone water at storage times of < 1 min and 3 h; * significant difference (p < 0.05) in comparison to organism viability test, Tukey-HSD

In the suspension test (Table 2), after the short storage time, the neutralized antiseptic agent was capable of significantly reducing the number of *Micrococcus luteus *CFU (p < 0.001; Tukey-HSD) and *Candida albicans *CFU (p < 0.001) in comparison to the organism viability test. After 3 h of storage, four of five neutralizations in the suspension test failed (*Staphylococcus epidermidis*, p < 0.001, *Micrococcus luteus *p < 0.001, *Candida albicans*, p < 0.001 and *Corynebacterium jeikeium*, p = 0.044).

**Table 2:** Suspension test for validation of neutralization of chlorhexidine digluconate.

Storage time	Test organism exposed to (name of the test)	*Staphylococcus * *aureus*	*Staphylococcus * *epidermidis*	*Micrococcus * *luteus*	*Corynebacterium * *jeikeium*	*Candida * *albicans*
<1 min.	neutralized antiseptic solution (neutralizer toxicity test)	1.82 ± 0.04	1.77 ± 0.03	0*	1.66 ± 0.09	1.06 ± 0.12*
	saline peptone water (organism viability test)	1.90 ± 0.05	1.84 ± 0.07	1.74 ± 0.03	1.79 ± 0.02	2.02 ± 0.03

3 h	neutralized antiseptic solution (neutralizer toxicity test)	1.80 ± 0.02	0.86 ± 0.14*	0*	1.44 ± 0.22*	0.13 ± 0.23*
	saline peptone water (organism viability test)	1.88 ± 0.06	1.81 ± 0.02	1.73 ± 0.05	1.77 ± 0.08	2.03 ± 0.02

Mean and SD of the number of CFU (log_10_) when exposed to the neutralized antiseptic solution or saline peptone water at storage times of < 1 min and 3 h; * significant difference (p < 0.05) in comparison to organism viability test, Tukey-HSD

**Figure 1:**
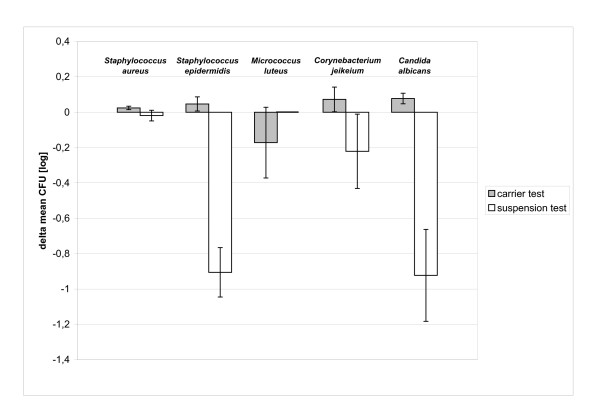
Delta of mean of CFU (log) between 3 h storage and immediate spread of neutralized antiseptic solution.

The results of the carrier test showed that no validation failed in the neutralizer effectiveness test after immediate spreading (Table 3). After 3 h of storage, the number of *Micrococcus luteus *CFU decreased (Figure 1) which resulted in a significant difference in comparison to the number of CFU in the organism viability test (p = 0.004).

**Table 3:** Carrier test for validation of neutralization of chlorhexidine digluconate.

Storage time	Test organism exposed to (name of the test)	*Staphylococcus * *aureus*	*Staphylococcus * *epidermidis*	*Micrococcus * *luteus*	*Corynebacterium * *jeikeium*	*Candida * *albicans*
<1 min.	neutralized antiseptic solution (neutralizer toxicity test)	1.84 ± 0.01	1.83 ± 0.04	1,61 ± 0.05	1.84 ± 0.01	2.02 ± 0.03
	saline peptone water (organism viability test)	1.90 ± 0.05	1.84 ± 0.07	1.74 ± 0.03	1.79 ± 0.02	2.02 ± 0.03

3 h	neutralized antiseptic solution (neutralizer toxicity test)	1.87 ± 0.01	1.87 ± 0.01	1.44 ± 0.14*	1.91 ± 0.08	2.10 ± 0.04
	saline peptone water (organism viability test)	1.88 ± 0.06	1.81 ± 0.02	1.73 ± 0.05	1.77 ± 0.08	2.03 ± 0.02

Mean and SD of the number of CFU (log_10_) when exposed to the neutralized antiseptic solution or saline peptone water at storage times of < 1 min and 3 h; * significant difference (p < 0.05) in comparison to organism viability test, Tukey-HSD

In four cases in the suspension test and one case in the carrier test, the number of surviving microorganisms decreased within the 3-hour storage period. Figure 1 shows the differences between the means for the 3-h stored samples and the immediate spread samples.

The highest decrease in cell number was found in the suspension test with *Candida albicans *(Δ of mean of CFU [log_10_] = -0.92, p = 0.004, t-test), followed by *Staphylococcus epidermidis *(Δ of mean of CFU [log_10_] = -0.91, p = 0.006). In spite of these significant differences in comparison to the organism viability test, the differences were not significant when compared with *Corynebacterium jeikeium*. Due to the fact that *Micrococcus luteus *was reduced to below the limit of detection within one minute of the contact time, no further reduction was detected after storage for 3 h. When a carrier was used, the CFU [log_10_] counts of *Micrococcus luteus *decreased within the 3-h storage period, however, this decrease was not statistically significant.

## Discussion

We were able to show for the first time that the validation of neutralization is strongly influenced by the amount of active agents, the amount of neutralizing agents, the storage time of the neutralized antiseptic before plating and by the chosen microorganism.

The need for neutralization validation as a basis of antimicrobial efficacy evaluations was emphasized by the fact that in studies without neutralizing agents in the sampling fluid, the efficacy of CHG was remarkably higher than in studies with neutralization [9,10]. A major limitation of many studies regarding the efficacy of antiseptic products is that the neutralization process is not specified at all. It is often not described if the active antimicrobial agents were neutralized, if the neutralization was validated and if the validation procedure followed the conditions of the efficacy evaluation [2].

One critical point in neutralization validation is the ratio between the amount of antiseptic agent and the amount of neutralizing agents available. In our study, 0.5 mL of antiseptic agents were mixed with 5 mL of broth containing the neutralizing agents, and 0.14 mL of antiseptic agents were mixed with 5 mL of broth containing the neutralizing agents in the carrier tests, this resulted in about three times more antiseptic solution in the suspension test than in the carrier test. After the short exposure time of < 1 min, neutralization failed with *Micrococcus luteus *and *Candida albicans *in the suspension test whereas neutralization was valid with all five test organisms in the carrier test. After 3 h storage of the neutralized antiseptic, the difference between the carrier tests and the suspension tests became more obvious. Neutralization failed in the suspension test with four of the five organisms tested, but only one failed (*Micrococcus luteus*) when the small amount of antiseptic solution was added in the carrier test. Whether the results depended on the type of test (carrier or suspension) could not be determined with this study design because equal amounts of the antiseptic solution were not compared in the two tests. There is some evidence in the literature which suggests a possible interaction between CHG and fibres such as cotton, therefore, we can not exclude the possibility that the swab material may have influenced the test results [20].

Based on our data it appears reasonable to conclude that validation of neutralization is acceptable for a carrier test if the experimental evidence is obtained from a suspension test and the amount of antiseptic solution in the carrier test is at least equal or less than that in the suspension test. Nevertheless, simulation of the efficacy test conditions as close as possible in the validation process is preferred.

Another critical point in neutralization validation is the storage time of neutralized antiseptic products. We were able to show that validation of neutralization is possible after a short storage time of the neutralized antiseptic product (< 1 min). If the neutralized antiseptic product, however, is stored for 3 h at 2 – 8°C, it may continue to kill microorganisms resulting in a lower number of surviving test organisms. If in an efficacy test of an antiseptic product, neutralization validation is performed with a very short storage time after sampling, and the efficacy test itself has a long storage time after sampling, this may well result in a lower number of surviving test organisms indicating better efficacy of the antiseptic product which could be classified as a false-positive efficacy result. Based on our data it is important that the storage time in the efficacy test is not longer than the storage time in the neutralization validation.

One parameter which has been overlooked so far, especially in the EN, is the impact of test organism choice on the outcome of neutralization and neutralization validation. To our knowledge this is the first study to show the importance of the type of microorganism in the validation process which is relevant for the subsequent efficacy evaluation. Based on our results it is not advisable to draw conclusions regarding the effectiveness of a neutralizer if different microorganisms are used in the validation process than in the efficacy evaluation. The efficacy of skin antiseptics is commonly determined using mixed skin flora on different skin sites (e.g. abdominal skin, groin, forehead, arms) [15,21]. If neutralization validation of a skin antiseptic is only determined using *Staphylococcus aureus*, the overall efficacy of the skin antiseptic could be overestimated because it is possible that other resident microorganisms such as *Micrococcus luteus*, which is more sensitive to residual CHG, will still be affected in the efficacy evaluation despite "valid neutralization".

In general the validation conditions should simulate the efficacy test conditions as closely as possible. With regard to all the challenge microorganisms used in this study, *Micrococcus luteus *was the most sensitive test organism and appears therefore to be appropriate for the sensitive detection of neutralizer validity.

## Conclusion

Valid neutralization during testing is essential for the scientific assessment of antiseptic efficacy in a defined contact time. Without valid neutralization false-positive efficacy data are likely. The design of the validation process should closely follow the design of the efficacy test. Attention should be paid in the validation process to the amount of antiseptic solution, the storage time and to the choice of appropriate and sensitive microorganisms.

## Competing interests

The first and the last authors are paid employees of Bode Chemie GmbH & Co. KG, Hamburg, Germany.

## Authors' contributions

MR designed the study, collected and analyzed the data and drafted the manuscript. GK participated in the study design, data analysis and helped to draft the manuscript. All authors read and approved the final manuscript.
